# Isoform-Specific Effects of Wild-Type *Ras* Genes on Carcinogen-Induced Lung Tumorigenesis in Mice

**DOI:** 10.1371/journal.pone.0167205

**Published:** 2016-12-02

**Authors:** Jamie D. Weyandt, John M. Carney, Elizabeth N. Pavlisko, MengMeng Xu, Christopher M. Counter

**Affiliations:** 1 Department of Pharmacology & Cancer Biology, Duke University Medical Center, Durham, North Carolina, United States of America; 2 Department of Pathology, Duke University Medical Center, Durham, North Carolina, United States of America; 3 Department of Radiation Oncology, Duke University Medical Center, Durham, North Carolina, United States of America; Case Western Reserve University, UNITED STATES

## Abstract

The gene *KRAS* is commonly mutated in lung cancer to encode a constitutively active and oncogenic protein that is well established to initiate and maintain lung tumorigenesis. However, the remaining wild-type KRAS protein, or the other family members HRAS and NRAS, can still be activated in the presence of oncogenic KRAS. Moreover, loss of any one of these three genes has been shown to increase the sensitivity of mice to the carcinogen urethane, which induces *Kras* mutation-positive early lung lesions. To determine the contribution of progressively disrupting *Hras* and *Nras* genes on urethane lung tumorigenesis, mice with different combinations of wild-type and null alleles of *Hras* and *Nras* were exposed with urethane and tumor burden was assessed. As previously reported, loss of one allele of *Hras* increased the sensitivity of mice to this carcinogen, and this effect was further exacerbated by the loss of the second *Hras* allele. However, loss of one or both alleles of *Nras* failed to alter tumor burden, either in the absence or presence of *Hras*, after exposure to urethane. Additionally, no obvious difference between lung lesions in mice with wild-type versus null alleles was detected, suggesting that wild-type Ras proteins may exert a tumor suppressive effects at the time of initiation, although other interpretations are certainly possible. In summary, these data suggest that in some genetic backgrounds inactivation of different wild-type *Ras* genes can have different effects on urethane-induced lung tumorigenesis.

## Introduction

Approximately one third of human cancers have an activating mutation in one of the three *RAS* genes; *HRAS*, *NRAS*, or *KRAS* [1]. RAS proteins function as molecular switches, alternating between inactive GDP-bound and active-GTP bound forms [2]. Conversion to the active GTP-bound state is greatly accelerated by guanine nucleotide exchange factors (GEFs) that promote the exchange of GDP for GTP, whereas GTPase activating proteins (GAPs) enhance GTP hydrolysis, reverting RAS proteins back to their inactive GDP-bound state [3]. Oncogenic mutations in these genes, typically at codons 12, 13, or 61, inactivate the inherent or GAP-stimulated GTPase activity of the enzyme, thereby rendering RAS constitutively active. In this state, chronic activation of downstream signaling pathways, including MAPK, PI3K, and RalGEF, results in uncontrolled cellular proliferation among other effects, driving tumor formation and growth [4].

*KRAS* is the most commonly mutated isoform in human cancers, and is well known to both initiate and maintain a wide spectrum of cancers, but especially pancreatic, colorectal, and lung cancers [5]. In regards to the latter, approximately 220,000 individuals in the United States are diagnosed with lung carcinoma each year, making it the second-most commonly diagnosed type of cancer [6]. Non-small-cell lung carcinoma (NSCLC) is the most prevalent type of lung cancer, and *KRAS* is the driving oncogene in about 20 to 30% of these cases [7]. As such, NSCLC is the cancer in which *RAS* mutations affect the highest number of individuals. Therefore, it is important to understand the role that RAS signaling plays in this disease.

Although oncogenic RAS proteins are well appreciated to drive tumorigenesis, the remaining wild-type family members can also be activated in the presence of the oncogenic proteins [8–12]. The consequences of this activation are complex. In some cases, particularly in models of early stage cancer, the wild-type isoforms inhibit oncogenic RAS-driven tumorigenesis [8, 13–15]. In contrast, in *KRAS*-mutation-positive cancer cell lines, wild-type RAS signaling instead promotes proliferation and tumorigenesis [9–13, 16]. These observations suggest that wild-type RAS proteins may have temporal, tissue, or other context-dependent effects on oncogenic RAS-driven tumorigenesis.

With regards to lung cancer, loss of both alleles of wild-type *Hras*, loss of both alleles of wild-type *Nras*, or loss of the remaining wild-type *Kras* allele, significantly increases the number of oncogenic *Kras*-driven lung tumors in mice exposed to the carcinogen urethane [13, 15, 17]. In agreement, the opposite experiment, namely increasing the expression of the remaining wild-type *Kras* allele, reduces lung tumorigenesis driven by oncogenic Kras [18]. These findings suggest that in early lung tumorigenesis induced by the carcinogen urethane, the wild-type *Ras* genes have tumor suppressive properties.

Since the loss of any of the wild-type *Ras* genes individually enhances urethane carcinogenesis, we explored whether progressive loss of an increasing number of *Ras* alleles would have an additive effect at promoting lung tumorigenesis. To this end, we generated littermates with different combinations of *Nras* and/or *Hras* null alleles. These mice were then treated with the carcinogen urethane to induce *Kras* mutation-positive lung lesions. We report here that, consistent with previous findings [13], loss of one allele of wild-type *Hras* enhanced lung tumorigenesis in mice treated with the carcinogen urethane, and that this effect was more pronounced when both alleles of *Hras* were inactivated. Interestingly however, loss of one or both alleles of wild-type *Nras* had no significant effects on the number of lung tumors that developed. Moreover, loss of wild-type *Nras* did not enhance the numbers of tumors observed in an *Hras*-null background. These results suggest the intriguing possibility that there are isoform-specific effects of wild-type *Ras* genes on urethane-induced lung tumorigenesis in some genetic backgrounds.

## Materials and Methods

### Generation of mice and genotyping

*Hras*^*-/-*^ and *Nras*^*-/-*^ mice were obtained from the NCI and Eugenio Santos (University of Salamanca, Spain). *Hras*^*+/-*^*;Nras*^*+/-*^ mice on a mixed 129/BL6 background were bred to obtain littermates with various combinations of native and null *Hras* and *Nras* alleles. Genomic DNA isolated from a total of 268 pups was genotyped for *Hras* and *Nras* wild-type and null alleles as previously described [19].

### Urethane carcinogenesis

9 to 14 mice per cohort at 7 to 9 weeks of age were intraperitoneally injected with 1 mg/g urethane (Sigma-Aldrich, dissolved in sterile PBS) as previously described [15]. Mice were then visually monitored for signs of pain, distress, or moribundity (sudden behavioral change, poor posture or ambulation difficulty, loss of hair coat condition, sudden activity change, painful facial expressions, neurological disorders, and cardiopulmonary disorders, according to the Duke University Institutional Animal Care and Use Guidelines) and weighed three times a week. Animals reaching these endpoints, or exhibiting at 15% reduction in weight were to be euthanized. However, no mouse exhibited signs of pain, distress, or moribundity or during the course of evaluation, and as such all mice in this study (barring one animal that was found dead for unexplained reasons) were humanely euthanized at the fixed time point of 11 months post-injection. Humane euthanasia was achieved by gradually replacing atmosphere air with CO_2_ followed by bilateral thoracotomy. This study was carried out in strict accordance with the recommendations in the Guide for the Care and Use of Laboratory Animals of the National Institutes of Health and under protocol A279-13-11 approved by the Institutional Animal Committee on Use and Care at Duke University.

### Quantification of lesions

Mice were euthanized at a fixed endpoint of 11 months after administration of urethane, the lungs were removed, and the number and size of surface tumors visible under a dissecting microscope at 10X magnification was documented without knowledge of the genotype. The left lung from each mouse was formalin-fixed, paraffin-embedded, and sectioned every 200 μm. Eight such sections were mounted on slides and H&E stained for pathologic analysis. Two pathologists (JMC and ENP), blinded to genotype, independently graded and counted the lesions in each section from 9 to 14 mice per cohort.

### Immunohistochemistry

Additional slides prepared from the above sectioning of the left lungs of mice exposed to urethane were subjected to epitope retrieval and stained with, anti-Ki67 (Thermo Scientific RM9106), anti-CC3 (Cell Signaling, D175), anti-P(Thr 202/Tyr 204)-Erk1/2 (Cell Signaling 4376), and anti-P(Ser473)-Akt (Cell Signaling 4060), anti-F4/80 (ABDserotec MCA497GA), and anti-CD3 (ThermoFisher RM9107) antibodies, followed by peroxidase-based detection with Vectastain Elite ABC Kits (Vector Labs) and counterstained with haematoxylin. Photographs were taken of 6 to 10 (10X) random fields in a blinded fashion. The tumor areas were determined from these photographs using the freehand selection tool in Image J (imagej.nih.gov). Color thresholding was applied to determine positive-staining areas, using the same parameters for each tumor image. Areas staining positive by these parameters were selected and the positive-staining area in pixels recorded. The percentage of positive-staining area was calculated by dividing the positive-staining area of the tumor in pixels by the total area of tumors in pixels.

### Statistics

Statistical Analyses were performed using GraphPad Prism v5 (GraphPad Software). A 2-sided, unpaired *t*-test was used to compare the number of lesions and levels of immunohistochemical staining between cohorts, and *p* values were calculated using the log-rank (Mantel-Cox) test.

## Results

### Loss of wild-type *Hras*, but not *Nras*, enhances the formation of urethane-induced lung tumors

**Fig 1:**
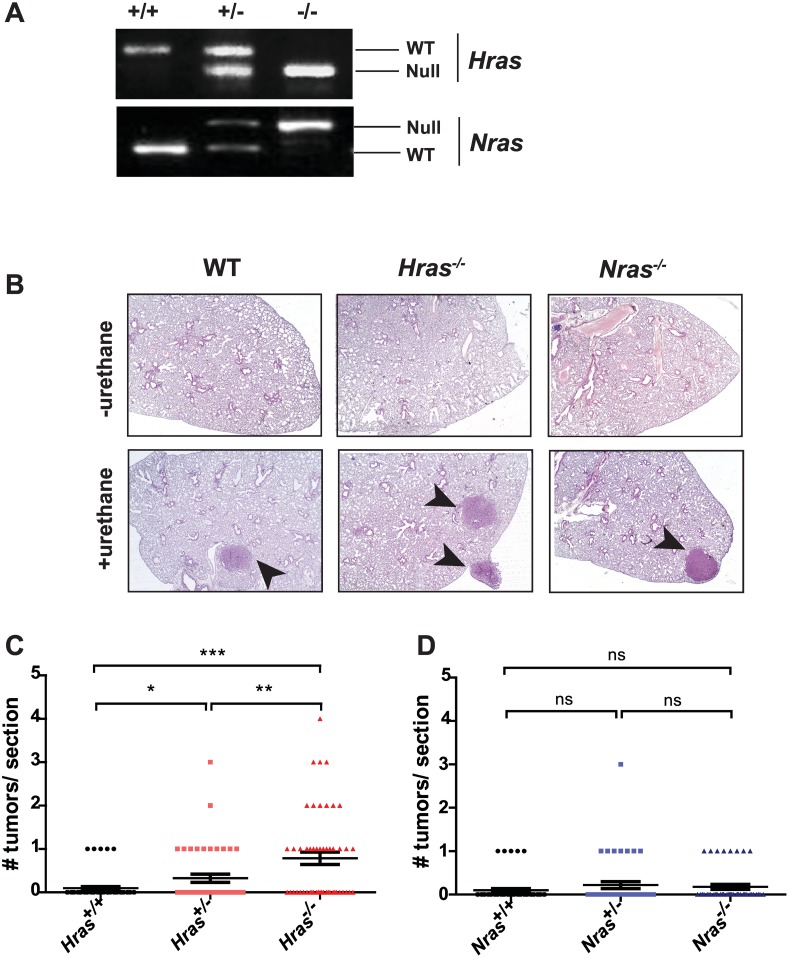
Loss of wild-type *Hras*, but not *Nras*, promotes formation of urethane induced lung tumors. (**A**) PCR-genotyping for *Hras* (*top panel*: wild-type (WT) band = 434 bp; null band = 336 bp) and *Nras* (*lower panel*: wild-type (WT) band = 146 bp; null band = 315 bp). (**B**) Representative H&E-stained lung tissue (magnification = 2X) from wild-type (WT), *Hras*^*-/-*^, and *Nras*^*-/-*^ mice (*top*) untreated and (*bottom*) 11 months after urethane administration. Arrowheads: lung lesions. (**C** and **D**) Number of lesions counted per H&E stained section from left lungs of mice 11 months after urethane administration in mice with the indicated combinations of wild-type (+) or null (-) alleles of (**C**) *Hras* or (**D**) *Nras* (bar: mean ± S.E.M.). ns: not significant. *p < 0.05. **p < 0.001. ***p < 0.0001. *n* = 4 to 6 sections from 9 to 14 mice per cohort.

To determine the effects of loss of progressively more wild-type *Ras* genes on carcinogen-induced lung tumorigenesis, mice lacking different combinations of wild-type *Hras* and *Nras* alleles were administered the chemical carcinogen urethane, which is well established to induced *Kras* mutation-positive lung adenomas and adenocarcinomas by 9 to 12 months [13, 15, 17]. More specifically, *Hras*^*+/-*^*;Nras*^*+/-*^ mice on a mixed 129/B6 background were interbred, generating littermates with one of the following 9 genotypes: *Hras*^*+/+*^*;Nras*^*+/+*^, *Hras*^*+/-*^*;Nras*^*+/+*^, *Hras*^*+/+*^*;Nras*^*+/-*^, *Hras*^*+/-*^*;Nras*^*+/-*^, *Hras*^*-/-*^*;Nras*^*+/+*^, *Hras*^*+/+*^*;Nras*^*-/-*^, *Hras*^*+/-*^*;Nras*^*-/-*^,*Hras*^*-/-*^*;Nras*^*+/-*^, and *Hras*^*-/-*^*;Nras*^*-/-*^. PCR amplification of genomic DNA isolated from of 268 pups was used to identify the status of the *Hras* and *Nras* alleles (*e*.*g*. Fig 1A), which revealed roughly the expected Mendelian ratios of the 9 genotypes (Table 1). The only exception was *Hras*^*-/-*^*;Nras*^*-/-*^ mice, which were observed at a lower than expected frequency (Table 1), as documented previously [19]. At 7 to 9 weeks of age, 9 to 14 littermates from each of the 9 aforementioned genotypes were injected intraperitoneally with urethane to induce *Kras*-mutation-positive lung lesions. These mice were euthanized 11 months later, and number and size of surface lung tumors visible at 10-fold magnification was determined.

**Table 1:** Distribution of genotypes from crossing *Hras*^*+/-*^*;Nras*^*+/-*^ mice.

Genotype	Expected ratio % (#)	Observed ratio % (#)
*Hras*^*+/+*^*;Nras*^*+/+*^	6.25 (16.75)	7.46 (20)
*Hras*^*+/-*^*;Nras*^*+/+*^	12.5 (33.5)	13.06 (35)
*Hras*^*+/+*^*;Nras*^*+/-*^	12.5 (33.5)	12.31 (33)
*Hras*^*+/-*^*;Nras*^*+/-*^	25.0 (67)	24.25 (65)
*Hras*^*-/-*^*;Nras*^*+/+*^	6.25 (16.75)	5.22 (14)
*Hras*^*+/+*^*;Nras*^*-/-*^	6.25 (16.75)	7.46 (20)
*Hras*^*-/-*^*;Nras*^*+/-*^	12.5 (33.5)	12.69 (34)
*Hras*^*+/-*^*;Nras*^*-/-*^	12.5 (33.5)	14.18 (38)
*Hras*^*-/-*^*;Nras*^*-/-*^	6.25 (16.75)	3.36 (9)

18 pairs of *Hras*^*+/-*^*;Nras*^*+/-*^ mice were bred to generate 268 pups. The percent and number of pups with each of the 9 possible genotypes (observed ratio) is show in comparison to the expected frequency based on Mendelian ratios (expected ratio).

As expected, urethane induced lung lesions in all the mice (*e*.*g*. Fig 1B). Consistent with previous studies [13], microscopic analysis revealed that *Hras*^*+/-*^*;Nras*^*+/+*^ mice, which have one *Hras* null allele, developed roughly three times more visible tumors than control *Hras*^*+/+*^*;Nras*^*+/+*^ mice. This effect was further exasperated by loss of the second wild-type allele, as *Hras*^*-/-*^*;Nras*^*+/+*^ mice developed, on average, more than twice as many tumors as *Hras*^*+/-*^*;Nras*^*+/+*^ mice (Fig 1C). In contrast to the progressive effect that the loss of wild-type *Hras* alleles had on lung tumorigenesis, there was no statistically significant difference in the number of lung tumors in *Hras*^*+/+*^*;Nras*^*+/-*^ or *Hras*^*+/+*^*;Nras*^*-/-*^ (Fig 1D) mice that contain either one or two *Nras* null alleles. Thus, in this mixed genetic background, the progressive loss of *Hras* alleles, but not *Nras* alleles, results in an increase in tumor burden upon exposure to the carcinogen urethane.

### Concomitant loss of wild-type *Hras* and *Nras* does not enhance the formation of urethane-induced tumors

**Fig 2:**
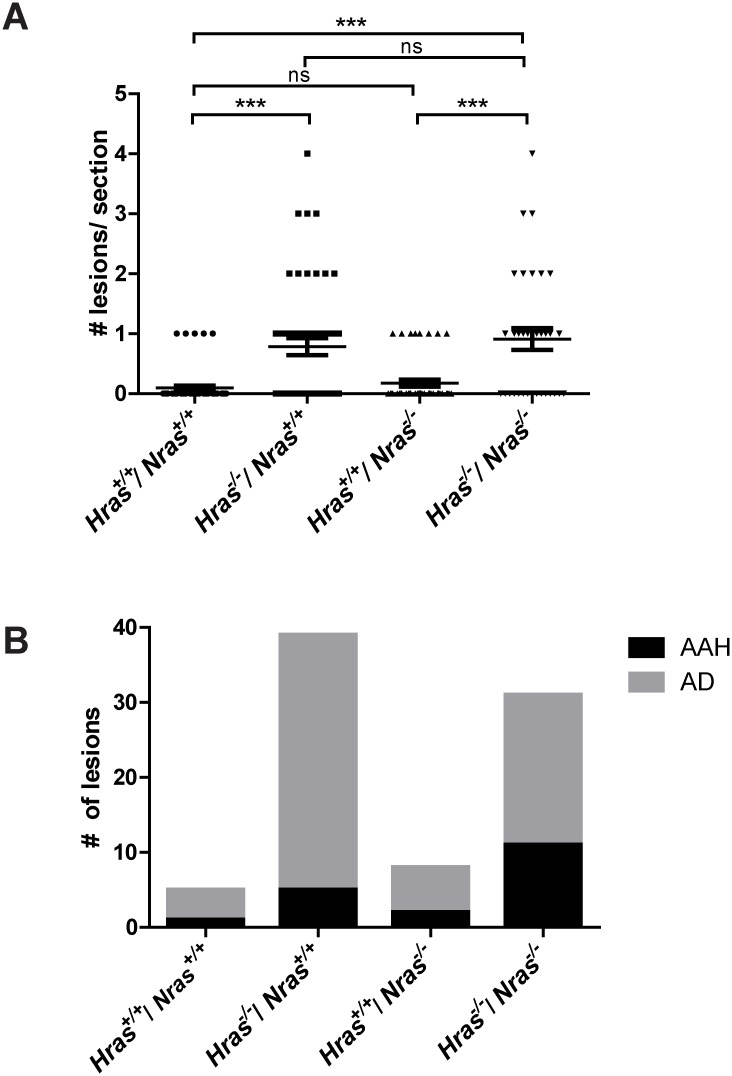
Effects of concomitant loss of wild-type *Hras* and *Nras* on urethane-induced tumorigenesis. (**A**) Number of lesions counted per H&E-stained section of lung tissue from urethane-treated mice with the indicated combinations of wild-type (+) or null (-) *Hras* and *Nras* alleles. (**B**) Number and grade of lesions from H&E-stained lung sections (bar: mean ± S.E.M.). AAH = atypical adenomatous hyperplasia. AD = adenoma. ns: not significant. ***p < 0.0001. *n* = 4 to 6 sections from 9 to 14 mice per cohort.

Previous studies have shown that loss of both wild-type *Hras* and wild-type *Nras* individually enhanced urethane-induced tumorigenesis in a dosage-dependent manner [13]. This suggests that loss of both of these isoforms could have an additive effect on promoting tumorigenesis in this model. To explore the effects of losing both *Hras* and *Nras* on urethane-induced tumors, the number of lung lesions that developed in double homozygous *Hras*^*-/-*^*;Nras*^*-/-*^ knockout mice was determined. These mice developed approximately the same number of tumors as *Hras*^*-/-*^*;Nras*^*+/+*^ mice lacking only *Hras* (Fig 2A). Thus, the enhanced tumorigenesis in mice lacking wild-type *Hras* is unaffected by the additional loss of wild-type *Nras* in this genetic background.

To assess if loss of progressively more *Ras* alleles alters tumor progression, tumors developing in *Hras*^*+/+*^*;Nras*^*+/+*^ control mice, *Hras*^*-/-*^*;Nras*^*+/+*^ mice lacking *Hras*, *Hras*^*+/+*^*;Nras*^*-/-*^ mice lacking *Nras*, or *Hras*^*-/-*^*;Nras*^*-/-*^ mice lacking both *Hras* and *Nras* were graded in a blinded fashion by 2 pathologists. All but one of the lesions were either atypical adenomatous hyperplasia (AAH) or adenoma (AD), both of which are early stages of lung tumorigenesis. One mouse (*Hras*^*-/-*^*;Nras*^*+/+*^) had a lesion graded as adenocarcinoma (not shown in figure). Comparing tumors from all 4 genotypes revealed no significant difference in the grade of lesions between these different genotypes (Fig 2B). Thus, loss of any of the tested combinations of wild-type *Hras* or *Nras* alleles did not alter the grade of lesions developing in the urethane model of lung tumorigenesis.

### Loss of wild-type *Hras* and *Nras* does not affect levels of proliferation, apoptosis, or downstream-signaling within urethane-induced tumors

**Fig 3:**
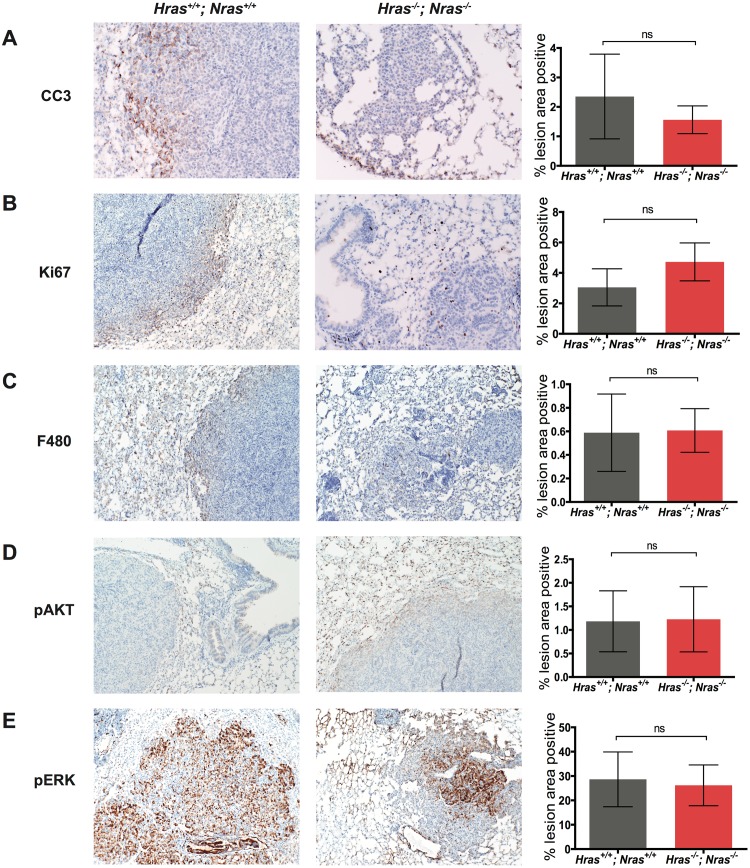
Immunohistochemical staining in urethane-induced lung lesions. (**A**) Cleaved caspase 3 (CC3), (**B**) Ki67, (**C**) F480, (**D**) phosphorylated AKT (pAKT), and (**E**) phosphorylated ERK (pERK) immunohistochemical staining (*left* panels, examples) and quantification thereof (*right*, graphs), based on the percentage of positively-staining tumor area calculated for 6–10 lesions from 3–5 mice with the indicated combinations of wild-type (+) or null (-) *Hras* and *Nras* alleles. Bar: mean ± S.E.M. ns: not significant.

To explore the effect of loss of wild-type *Ras* genes on tumor characteristics, 6 to 10 lung lesions from urethane-treated mice with the two extreme genotypes, namely *Hras*^*+/+*^*;Nras*^*+/+*^ versus *Hras*^*-/-*^*;Nras*^*-/-*^, were stained for markers of apoptosis (cleaved caspase 3), proliferation (Ki67), inflammation (F480), and Ras signaling (phosphorylated ERK and AKT). No significant differences in immunohistochemical staining using antibodies against cleaved caspase 3 (Fig 3A), Ki67 (Fig 3B), or F480 (Fig 3C) were observed between these two cohorts. Furthermore, the average levels of phosphorylated AKT (Fig 3D) and ERK (Fig 3E) immunostaining within the lesions were also similar between these two cohorts. Similarly, loss of just Hras had no measurable effect of MAPK signaling, as assessed by the levels of phosphorylated ERK (S1A Fig).

### Loss of wild-type *Hras* and *Nras* in combination increases the number of lymphoid aggregates in the lungs of urethane-treated mice

**Fig 4:**
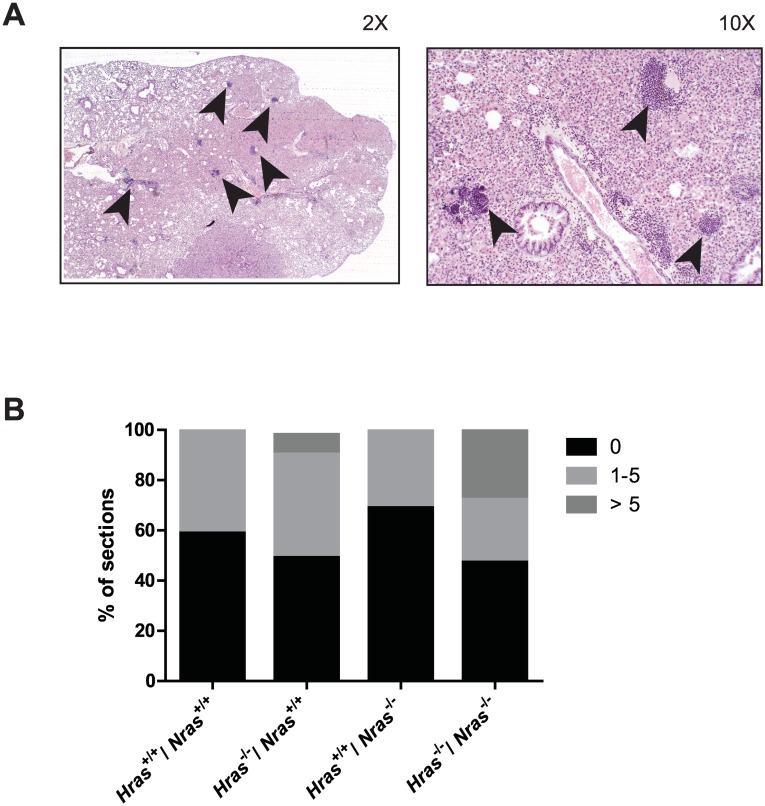
Loss of wild-type *Hras* alone or in combination with loss of wild-type *Nras* promotes lymphoid infiltration in the lungs of urethane-treated mice. (**A**) Representative photograph at 2X (*right*) and 10X (*left*) magnification of H&E-stained lung tissue containing lymphoid aggregates (indicated by arrowheads). (**B**) Quantification of the percentage of sections from mice with the indicated combinations of wild-type (+) or null (-) *Hras* and *Nras* alleles in which lymphoid aggregates are binned as no aggregates, 1–5 aggregates, or >5 aggregates. *n* = 4 to 6 sections from 9 to 14 mice per cohort.

Pathological examination of lung sections revealed an aggregation of lymphoid cells in mice exposed to urethane (Fig 4A). To assess whether disruption of either or both *Hras* and *Nras* alleles influenced this lymphoid aggregate phenotype, two pathologists determined the number of lymphoid aggregates in each section of lungs isolated from the 4 genotypes *Hras*^*+/+*^*;Nras*^*+/+*^, *Hras*^*-/-*^*;Nras*^*+/+*^, *Hras*^*+/+*^*;Nras*^*-/-*^, and *Hras*^*-/-*^*;Nras*^*-/-*^. Binning these data into groups of no aggregates, 1–5 aggregates, or greater than 5 aggregates per section revealed that the number of lymphoid cell clusters were higher in *Hras*^*-/-*^*;Nras*^*+/+*^ mice, and even more prevalent in *Hras*^*-/-*^*;Nras*^*-/-*^ mice (Fig 4B). We note here this was not associated with changes in F480 staining (Fig 3C). Staining for cluster of differentiation (CD3) was also similar between *Hras*^*+/+*^*;Nras*^*+/+*^, *Hras*^*-/-*^*;Nras*^*+/+*^, and *Hras*^*-/-*^*;Nras*^*-/-*^ genotypes (S1B Fig). Thus, loss of wild-type *Hras* enhances the lymphoid response in the lungs to the chemical carcinogen urethane, and this response is further augmented by the additional loss of wild-type *Nras*.

## Discussion

We demonstrate that loss of wild-type *Hras* enhances carcinogen-induced lung tumorigenesis in mice, in agreement with previous findings [8, 13]. This effect was not, however, observed in mice lacking wild-type *Nras*, nor did loss of *Nras* increase the sensitivity of *Hras*^*-/-*^ mice to urethane. Such a finding presumably reflects a difference in either the expression or function of these two Ras isoforms. We note that loss of *Nras* had previously been shown to yield more lung tumors in mice treated with urethane [13]. One possible explanation for this difference is that the experiments described here employed a mixed genetic background while the aforementioned study use a pure 129 strain. Indeed, strain background is well established to greatly influence the susceptibility of mice to urethane-induced tumorigenesis [20]. More specifically, the mix background of the study described here was less sensitive to urethane carcinogenesis, which may mask effects due to disrupting *Nras* alleles. Nonetheless, in this background loss of *Hras* promoted *Kras*-driven lung tumorigenesis, while loss of *Nras* had no measureable effect.

Levels of Ras signaling, as measured by pAKT and pERK immunostaining, were similar in the lesions between mice with wild-type versus null *Hras* and *Nras* alleles. These lesions also had similar staining patterns for markers of proliferation, apoptosis, and inflammation. It is therefore possible that the effect of losing wild-type *Hras* occurs before the tumors develop, perhaps at the stage of initiation, and as such would not manifest a phenotype in established lesions. For example, loss of wild-type *Hras* may alter lung development, increasing the number of the cell types susceptible to urethane carcinogenesis. Alternatively, losing *Hras* in the stroma could affect tumorigenesis without altering tumor cell signaling. Finally, we recognize that there may yet be differences in signaling or markers of cellular features like proliferation, apoptosis or inflammation, but the effects are just too subtle to detect by immunohistochemical staining. Further studies are needed to determine the exact mechanisms by which loss of *Hras* promotes the growth of carcinogen-induced lung lesions.

Finally, lymphocyte infiltration was observed in *Hras* knockout mice exposed to urethane, a phenotype more pronounced upon the additional loss of the *Nras* gene. Aggregates of lymphoid tissue in the lung are most often in response to infection, but occasionally are seen in cases of NSCLC [21]. Knockdown of wild-type Nras has also been shown to alter pathways related to immune responses [22], and *Hras*^*-/-*^*;Nras*^*-/-*^ mice are unable to mount an immune response to parasitic infection, due to hampered Th1 immunity [23]. Taken together, these results suggest a relationship between the loss of wild-type Ras and an immune response, although whether this is related to urethane carcinogenesis, and if so how, remains to be determined.

In summary, we demonstrate that different wild-type Ras isoforms can have different effects on urethane carcinogenesis, adding important insight into the relationship between wild-type Ras proteins and early tumorigenesis.

## Supporting Information

S1 Fig Immunohistochemical staining in urethane-induced lung lesions. (**A**) Phosphorylated ERK (pERK) and (**B**) cluster of differentiation 3 (CD3) immunohistochemical staining and quantification of percentage of positively-staining tumor area calculated for 6–10 lesions from 4–6 mice of the indicated combinations of wild-type (+) and null (-) *Hras* and/or *Nras* alleles. bar: mean ± S.E.M. ns: not significant.
(EPS)
